# Effects of Ligand Binding on the Mechanical Properties of Ankyrin Repeat Protein Gankyrin

**DOI:** 10.1371/journal.pcbi.1002864

**Published:** 2013-01-17

**Authors:** Giovanni Settanni, David Serquera, Piotr E. Marszalek, Emanuele Paci, Laura S. Itzhaki

**Affiliations:** 1 Physics Department, Johannes Gutenberg University, Mainz, Germany; 2 MRC Cancer Cell Unit, Hutchison/MRC Research Centre, Cambridge, United Kingdom; 3 Department of Mechanical Engineering and Materials Science, Duke University, Durham, North Carolina, United States of America; 4 School of Molecular and Cellular Biology, University of Leeds, Leeds, United Kingdom; 5 University of Cambridge Department of Chemistry, Cambridge, United Kingdom; University of Missouri, United States of America

## Abstract

Ankyrin repeat proteins are elastic materials that unfold and refold sequentially, repeat by repeat, under force. Herein we use atomistic molecular dynamics to compare the mechanical properties of the 7-ankyrin-repeat oncoprotein Gankyrin in isolation and in complex with its binding partner S6-C. We show that the bound S6-C greatly increases the resistance of Gankyrin to mechanical stress. The effect is specific to those repeats of Gankyrin directly in contact with S6-C, and the mechanical ‘hot spots’ of the interaction map to the same repeats as the thermodynamic hot spots. A consequence of stepwise nature of unfolding and the localized nature of ligand binding is that it impacts on all aspects of the protein's mechanical behavior, including the order of repeat unfolding, the diversity of unfolding pathways accessed, the nature of partially unfolded intermediates, the forces required and the work transferred to the system to unfold the whole protein and its parts. Stepwise unfolding thus provides the means to buffer repeat proteins and their binding partners from mechanical stress in the cell. Our results illustrate how ligand binding can control the mechanical response of proteins. The data also point to a cellular mechano-switching mechanism whereby binding between two partner macromolecules is regulated by mechanical stress.

## Author Summary

Here we use molecular dynamics simulation to compare the mechanical properties of the 7-ankyrin-repeat oncoprotein Gankyrin in isolation and in complex with binding partner S6-C. Tandem repeat proteins like Gankyrin comprise tandem arrays of small structural motifs that pack linearly to produce elongated architectures. They are elastic, mechanically weak molecules and they unfold and refold repeat by repeat under force. We show that S6-C binding greatly increases the resistance of Gankyrin to mechanical stress. The enhanced mechanical stability is specific to those ankyrin repeats in contact with S6-C, and the localized nature of the effect results in fundamental changes in the way the protein responds to force. Thus, the forced unfolding of isolated Gankryin involves a diverse set of pathways with a preference for a C- to N-terminus unfolding mechanism whereas this diversity is reduced upon complex formation with the central repeats, which are those most tightly bound to the ligand, tending to unfold last. Our study shows how stepwise unfolding can buffer repeat proteins and their binding partners from mechanical stress in the cell. It also points to a mechano-switching mechanism whereby binding between two partner macromolecules is regulated by mechanical stress.

## Introduction

Tandem repeat proteins, also known as solenoid proteins, are a special class of proteins comprising tandem arrays of small structural motifs (20–40 residues) that pack in a roughly linear fashion to produce elongated, superhelical architectures, thereby presenting extended surfaces that act as scaffolds for molecular recognition. Examples include ankyrin, tetratricopeptide, HEAT and leucine rich repeats. Their structures are characterized by short-range interactions between residues either within a repeat or in adjacent repeats. As such they contrast with globular proteins, which are stabilized by many sequence-distant interactions that frequently result in complex topologies, and with polyproteins like titin, in which independently folded domains are covalently linked in tandem arrays but without significant non-covalent interactions between the individual domains. It is thought that the lack of sequence-distant contacts affords repeat proteins a high degree of flexibility and elasticity [1], [2], and atomic force microscopy (AFM) studies have identified certain unique properties that underlie this spring-like mechanical behavior [3]–[5], [6]. However, the relationship between spring and scaffold functions of repeat proteins is not understood and requires a determination of the mechanics of these proteins upon ligand binding.

**Figure 1:**
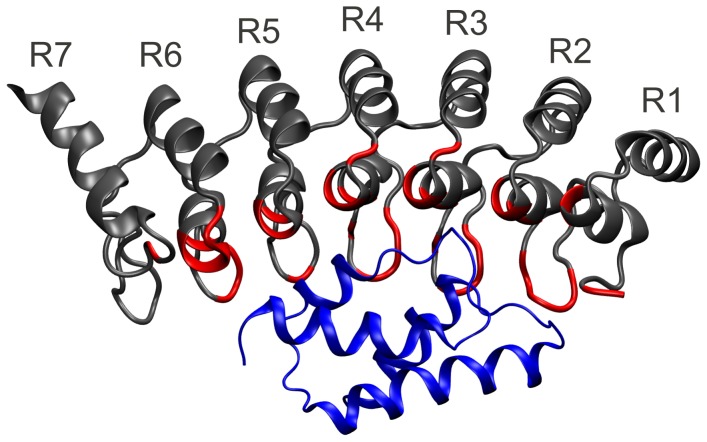
Cartoon representation of Gank-S6-C complex structure after the equilibration phase of the MD simulation. Gank is shown in grey and S6-C in blue. The residues of Gank with atoms closer than 5 Å to S6-C are shown in red.

Gankyrin is an oncoprotein that is overexpressed in hepatocellular carcinomas [7]. It belongs to the ankyrin repeat family of proteins, which are involved in numerous protein-protein interactions and which have been postulated to be the spring elements in mechanotransduction [8]. Each ankyrin repeat forms a β-turn followed by two antiparallel α-helices and a loop. Gankyrin binds the S6 ATPase subunit of the 19S regulatory particle of the 26S proteasome and it enhances the degradation of the tumour suppressors pRb and p53. The interaction of Gankyrin with S6 C-terminal domain (S6-C) is typical of repeat protein molecular recognition in that the whole length of Gankyrin is used to create an extended surface for binding [9] (Figure 1). All but the C-terminal ankyrin repeat of Gankyrin (repeat seven) make contacts with S6-C. The interaction involves complementary charged residues on the two proteins that form several positively and negatively charged patches along the elongated interface. The latter comprises residues from the β-turns and the N-terminal helices of repeats 1–6 of Gankyrin.

An important process in mechanotransduction is mechano-switching. For example, force has the potential to partially unfold proteins, shutting off or triggering biochemical reactions by disrupting binding motifs or exposing cryptic binding sites. Force modulates a protein's free energy surface; a small force does not necessarily abolish the native minimum but may cause the breakage of non-covalent bonds or, conversely, activate catch bonds that bind more tightly with force [10]. Likewise, binding can affect the mechanical response of proteins to external stress, as shown for Dihydrofolate Reductase [11], Im9 [12] and protein G [13], [14]. Here we use atomistic molecular dynamics simulations to compare the mechanics of Gankyrin in isolation and in complex with S6-C. The results show how the ligand affects every aspect of Gankyrin's behavior, including the order of repeat unfolding and the nature of partially unfolded intermediate states, the forces required and the work transferred to the system to unfold the whole protein and its parts. Gankyrin shows a unique behavior that may be prototypical of repeat proteins. In the absence of the S6-C ligand, there are many different mechanical unfolding pathways accessible to Gankyrin with a preference for a C- to N-terminus mechanism. In contrast, in the presence of S6-C the central repeats of Gankyrin, which make the most contact with the ligand, are stabilized and consequently the number of accessible unfolding pathways is reduced. Thus, the outermost repeats of Gankyrin unfold at low forces whereas the central repeats preserve their structure and binding interactions and only rupture at higher forces. The different force regimes under which Gank unfolds when bound to S6-C might have relevance in the mechanical control of protein-protein interactions in the cell and this behavior could be exploited in future to design new protein mechano-switches.

## Materials and Methods

### Molecular dynamics simulations

Molecular dynamics simulations were performed using the CHARMM program [15], [16] with the charmm19 united atom force field. The effect of the aqueous environment was modeled with the fast analytical continuum treatment of solvation (FACTS) algorithm [17]. An implicit solvent model was preferred to explicit ones because an atomistic representation of even a single hydration layer of an extended protein conformation would require an immense computational effort; moreover, implicit solvents relax instantaneously, which reduces artifacts when the protein is pulled fast. The previous application of this methodology to Gank [6] gave results that agreed with experimental measurements, which validates our choice of implicit solvation model. Initial atomistic models for Gank and Gank-S6-C complex were obtained from pdb structures 1qym [18] and 2dwz [9], respectively. The residues of Gank in both systems were renumbered starting from the first resolved residue, which is Cys4 (i.e., Cys 4 in the pdb file is named Cys 1 in the present manuscript, and the following residues are renamed accordingly). The number of resolved residues in 1qym is 223, whereas 226 residues of Gank were resolved in 2dwz. The three extra residues are at the C-terminus of Gank in the complex and they have been retained in the simulations. Langevin dynamics at 300 K with a friction coefficient of 1 ps^−1^ and an integration timestep of 2 fs was used. After 100 ps of equilibration at 300 K, the Gank constructs were subjected to steered molecular dynamics, whereby a spring with elastic constant set to 20 pN/Å was attached to the N-terminal main-chain nitrogen and to the C-terminal carbonyl carbon and its equilibrium distance was increased at constant velocity to simulate pulling. This resulted in an equal force being applied to both terminal atoms, along the vector joining them and in the direction of increasing distance. Pulling speeds of 0.05 Å/ps and 0.01 Å/ps were used. At pulling speed of 0.01 Å/ps, twelve independent simulations of isolated Gank and twelve of the Gank-S6-C complex were performed with different initial velocities starting from conformations sampled during equilibration. Each simulation was 80 ns long, which allowed for the complete stretching of Gank. Conformations were saved every 4 ps for analysis. At pulling speed of 0.05 Å/ps 24 and 16 simulations were performed for the Gank-S6-C complex and for the isolated Gank, respectively. Each simulation was 16 ns long and a snapshot was saved every 1 ps for analysis.

### Identification of force peaks

**Figure 2:**
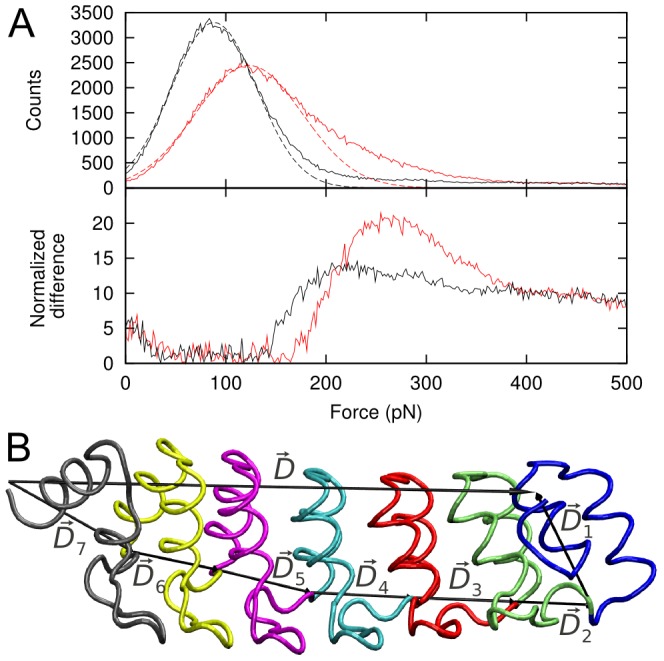
Distribution of the external force measured along the simulations. (A) Upper panel: Distribution of the force for isolated Gank (black solid line) and Gank-S6-C complex (red solid line). The peaks of the distributions were fitted to a Gaussian (dashed lines). Lower panel: Difference between the actual distribution and the fitted Gaussian, normalized using the error on the actual distributions. Peaks in the difference (at 200 pN and 250 pN, for isolated Gank and Gank-S6-C complex, respectively) were taken as thresholds to identify unambiguously peaks in the force-extension profiles. (B) Cartoon representation of the ankyrin repeats of Gank from N- to C-terminus are colored blue, green, red, cyan, magenta, yellow and dark grey, respectively. The end-to-end vector 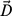 and its repeat components 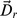 are indicated with black arrows.

**Figure 3:**
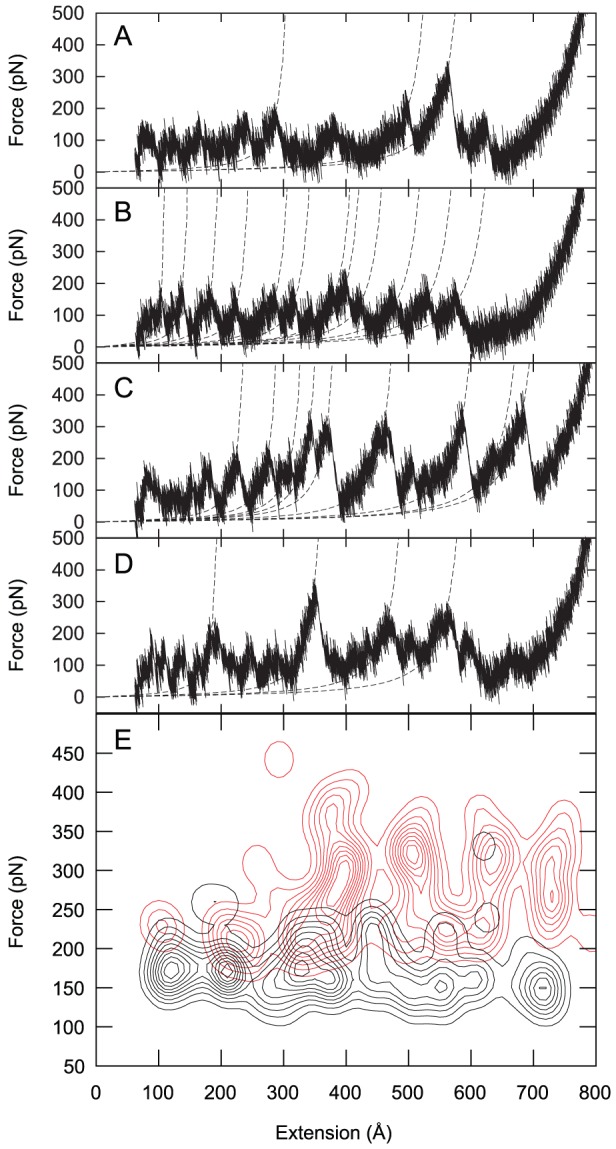
Force-extension profiles from representative simulations. Representative profiles are shown for isolated Gank (A)–(B) and for Gank-S6-C complex (C)–(D). The peaks were fitted to a WLC model with a persistence length of 0.38 nm (dashed lines). (E) Probability distribution of the force peaks on the extension-force plane of uncomplexed Gank (black) and Gank-S6-C complex (red). The distribution was obtained by a kernel density estimation analysis of the peak coordinates from all the sampled force-extension profiles, with a Gaussian bandwidth of 20 Å and 20 pN. The figure was prepared using Octave [37].

The distribution of the force measured along the simulations at 0.01 Å/ps resembles a normal distribution with a long tail in the large force regions (Figure 2A, top). The force distribution for the isolated Gank is narrower and peaks at lower values than does the distribution for the complex Gank-S6-C. The main part of the distribution resembles a Gaussian, possibly due to the large number of force-field parameters contributing to it. Thus, this part of the distribution was fitted to a normal distribution and the deviation between the actual distribution and the fit was determined (Figure 2A bottom). Force peaks in the force-extension profiles (Figure 3) reaching values where the deviation is the largest were retained as unambiguous peaks (i.e. values larger than 200 pN and 250 pN for the Gank and the Gank-S6-C simulations, respectively). Lower force peaks are not easily separable from the noise and were therefore discarded. Similar quasi-normal distributions were obtained at pulling speeds of 0.05 Å/ps, however, the number of force peaks above the noise level was very small and the force peak analysis was therefore not carried out.

### Measurement of work

The work transferred [19], [20] by the external force to unfold the protein was measured by integrating 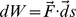 along the simulations, where 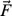 is the external force and 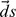 is the change in the vector connecting the two atoms where the force is applied (the N and C termini of the protein). This procedure is equivalent to measuring the area under the force-extension profiles (Figure 3). Because the data were saved at discrete time points, operatively we defined the work transferred by the external force up to time 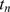 as:
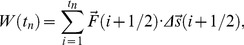
(1)where the sum runs along the saved time frames of the simulation, 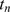 is the elapsed simulation time at frame *n*, 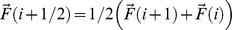 is the average force measured in two successive frames and 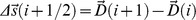 is the change in the end-to-end vector (connecting the backbone C atom at the C-terminus of Gank to the N atom at the N terminus) between two successive frames. Contributions from the individual ankyrin repeats are readily obtained by decomposing the 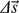 vector into its repeat components (see also Figure 2B):
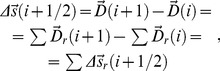
(2)where 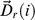 is the end-to-end vector of repeat *r* at frame *i* and 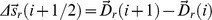 is the corresponding change in two successive frames. The individual repeat contributions of the work are then:
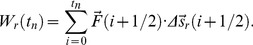
(3)A few examples of the behavior of 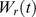 in selected runs are shown in Figure S1. We would like to emphasize that, whereas the assignment of force peaks to particular repeat unfolding events may sometimes be problematic because of the broadness of the peaks and noisy nature of the force extension profiles, the repeat component of the work is in contrast a well-defined and robust quantity that we can use instead to report on the forces needed to unfold the repeats *via* the integration step in Eq. 3.

### Monitoring repeat unfolding

The C_α_C_β_-atom root-mean-square deviation (RMSD) of each ankyrin repeat of Gank from the native x-ray conformation was monitored along the simulations. Typically, the RMSD of a single repeat remains smaller than 5 Å for a certain time and then it displays an abrupt rise due to repeat unfolding, quickly reaching values of 20–25 Å when fully extended. Thus, in each simulation and for each repeat, the unfolding midpoint was set as the time point when the RMSD reached 10 Å. Different unfolding RMSD thresholds (from 7 Å to 15 Å) did not significantly change the average repeat unfolding time and the statistics of the repeat unfolding sequences at either unfolding speeds. On the 0.01 Å/ps pulling speed simulations a contact analysis has been performed. Pairs of residues defined a native contact when the distance between their C_α_ atoms was smaller than 8 Å in the native structure. Only those pairs more than three residues apart in the sequence were considered. The lifetime of a contact was defined as the last time the contact was observed along the simulations. The identification of non-native contacts is based on the concept of recurrence, defined in S.I. The definition of recurrent contacts translates broadly to those contacts that could be observed consistently in at least a continuous 0.4-ns-long stretch of trajectory. Non-native contacts are recurrent contacts that are not present in the native state.

### Kernel density estimation

This technique is used to provide approximations to probability density distributions from a discrete sample of data points. Each point of the set is replaced by a Gaussian function with a fixed width. The total approximated probability density distribution for the data set is then the sum of all the Gaussian functions after normalization. The width of the Gaussian function is chosen on the basis of the expected error on the data values. The technique is used in place of a histogram of the data points, because it provides smooth distributions.

### Monitoring the Gank-S6-C interaction

The residues of Gank involved in binding to S6-C were defined as those that had at least one atom at a distance of less than 5 Å from any S6-C atom. The native binding residues were defined as those that were involved in binding in more than 80% of the equilibration trajectory. The residue binding lifetime was defined as the last time residues were observed to be involved in binding along the simulations, averaged over the different runs.

## Results

### Differences in the stepwise mechanical unfolding of uncomplexed and complexed Gankyrin

**Figure 4:**
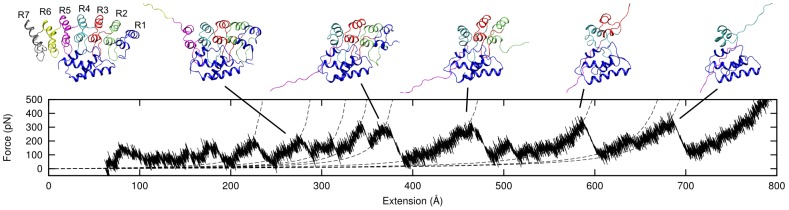
Representative snapshots from one of the simulations of complexed Gank. Snapshots were taken corresponding to the indicated peaks in the force-extension profile. The starting structure is shown on the left. The first peaks at extensions below 300 Å, show small forces and correspond to the unfolding of ankyrin repeats 7 and 6. Peaks at extensions larger than 300 Å show larger forces and correspond to the unfolding of the repeats with extensive contacts to SC-6 (i.e. R1–5); central repeats 3 and 4 are the last to unfold in this simulation.

Gank (pdbid 1qym [18]) and S6-C-bound Gank (pdbid 2dwz [9]) were first equilibrated and then subjected to constant speed steered molecular dynamics simulations using the program CHARMM [15], [16] with the united atom force field (param19) and FACTS [17] as model for implicit treatment of solvent (see “Materials and Methods” for further details about the simulations). During the pulling of Gank to the fully extended state the force exerted on the protein ends was recorded. In AFM experiments on polyproteins the peaks in the force-extension profiles generally correspond to the unfolding of single monomeric units [21], [22] and the increase in contour length between peaks corresponds to the unfolding length of a monomer; for ankyrin-repeat proteins [3], [5], [23], the peaks in the force-extension profiles generally correspond to the unfolding of single repeats and the increase in contour length between peaks corresponds to the unfolding length of a single repeat (105–120 Å), although contour length extensions of 40–70 Å corresponding to the unfolding of half a repeat have also been observed in the cases of Gank [6], AnkyrinR [3], and NI6C [5]). In the present simulations, only the lower pulling speed produced force-extension profiles in which a sufficient number of clear force peaks could be identified above the noise level (see “Materials and Methods” for further details about identification of force peaks). Representative force-extension profiles at the low pulling speed are shown in Figure 3A–D. The regions of the force-extension profile preceding the peaks were fitted separately to a worm-like chain (WLC) model with fixed persistence length of 3.8 Å, as in our earlier work [6]. The contour length and the breaking force for each peak were also measured. Representative snapshots from one of the simulation of the complexed Gank provide a pictorial description of the unfolding process (Figure 4).

**Figure 5:**
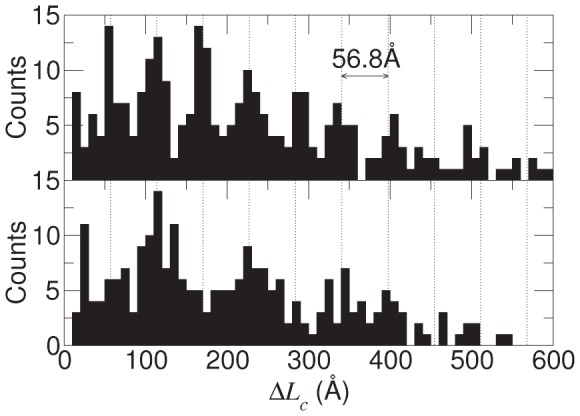
Histogram of the contour length differences of the force peaks (from the fit to the WLC model) observed in the force-extension profiles. Differences are computed between all possible pairs of peaks in the same simulation run (i.e., not only between adjacent peaks). Data for isolated Gank and for Gank-S6-C complex are shown in the upper and lower panels, respectively. Peaks in the histogram, corresponding to the most common changes in contour length after a force peak, show marked periodicity. Dashed lines are drawn to highlight the length L = 56.8 Å and 2 L of the periods observed in the data, 2 L corresponding to the contour length of one fully extended ankyrin repeat.

Analysis of the histogram of the contour length extensions shows that, for the uncomplexed Gank, the peaks are separated by distances that are multiples of L = 56.8 Å (Figure 5, top), corresponding to the unfolding length of half an ankyrin repeat [3], [5], [23]; a 2 L periodicity is also apparent. In the case of the Gank-S6-C complex, the 2 L periodicity is more evident and the peaks are most frequently separated by distances that are multiples of 2 L (Figure 5, bottom). Thus, the unfolding events in the uncomplexed Gank frequently involve half of a repeat (usually one of the two helices comprising the ankyrin motif), whereas in the Gank-S6-C complex the unfolding events more frequently involve a whole repeat. Our simulations are therefore in agreement with our previous AFM experiments on uncomplexed Gank.

### Higher force peaks are observed for complexed than for uncomplexed Gankyrin

As well as the differences in the step size of repeat unfolding described above, differences in the unfolding forces of uncomplexed *versus* complexed Gank, are also observed (Figure 3E). In uncomplexed Gank the force peaks are typically between 100 pN and 250 pN, and the majority of the peaks are observed at total extensions of less than 400 Å. In the case of the Gank-S6-C complex, the force peaks lie in the range of 200 pN and 400 pN and with total extensions frequently larger than 300 Å. The scarcity of force peaks at total extensions below 300 Å in the complex and above 400 Å in the uncomplexed Gank is not due to a lack of unfolding events in these ranges but rather it is due to the forces peaks lying below the noise level. Furthermore, in uncomplexed Gank, the force is roughly independent of contour length. In contrast, in the case of the Gank-S6-C complex those few peaks observed at total extensions below 300 Å, corresponding to the unfolding of repeats 7 and 6 (see below), have forces similar to those found in uncomplexed Gank, whereas peaks at total extensions above 300 Å show larger forces. Figure 3E shows that for both uncomplexed and complexed Gank the force peaks cluster at values of the extension that are separated approximately by the contour length of a single ankyrin repeat (∼110 Å), as discussed above), although the distribution is not sharp and intermediate values of the extension are also observed.

### Larger work is transferred to complexed Gankyrin compared with uncomplexed Gankyrin

**Figure 6:**
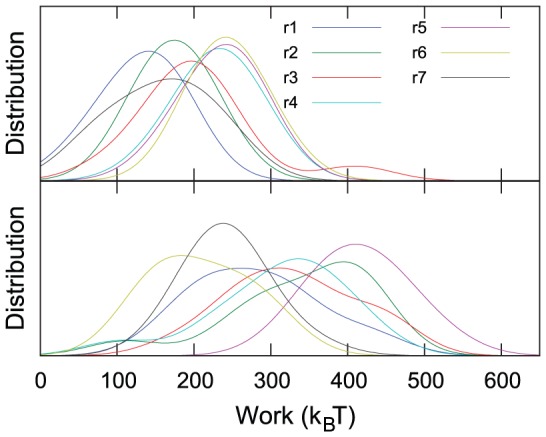
Distribution of the repeat components of the transferred work at 0.01 Å/ps pulling speed after full extension of Gank is reached (at t = 64 ns). The distribution is obtained by a kernel density estimation analysis of the work data with a Gaussian bandwidth of 50 k_B_T. Data for isolated Gank and Gank-S6-C complex are shown in the upper and lower panels, respectively.

The work transferred by the external force to unfold Gank was measured along the simulations (see “Materials and Methods” for a detailed description of this calculation), together with the repeat components of this work (Figure 6). These calculations do not require the identification of the force peaks, and consequently they could be performed at both of the pulling speeds used here. At the pulling speed of 0.01 Å/ps, significantly more work was needed to completely stretch (i.e. after 64 ns of simulations) the complexed Gank (2100±100 k_B_T) than the uncomplexed Gank (1400±100 k_B_T). Similarly at 0.05 Å/ps pulling speed, the work transferred for complete stretching was 3200±200 k_B_T and 2300±100 k_B_T for complexed and uncomplexed Gank, respectively. These values are of the same order of magnitude as values measured experimentally for other biological macromolecules [24], [25]. The larger work transferred at the higher pulling speed is expected due to the larger fraction of energy irreversibly dissipated in the faster process. At both pulling speeds, all of the repeats, with the exception of repeat 6, required significantly more work to unfold when Gank was bound to S6-C (t-test at 1%-significance level, Figure 6, and Table S1 and Table S2). Repeat 1 showed the largest change upon complexation, with about twice the work required for unfolding in the complex compared with isolated Gank (Table S1 and Table S2). The unexpected increase in the work required to unfold repeat 7, which does not directly contact S6-C, may be due to the larger length of this repeat (by 3 residues) in the complexed Gank construct compared with the uncomplexed Gank construct.

### The sequence of unfolding events is different in complexed *versus* uncomplexed Gankyrin

**Figure 7:**
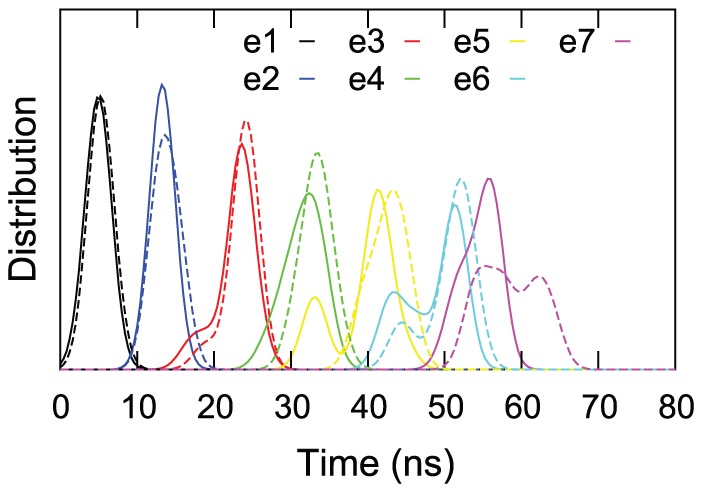
Unfolding times of individual ankyrin repeats of Gank at 0.01 Å/ps pulling speed. Distributions of successive repeat unfolding times (e1 corresponding to the first unfolding event) in isolated Gank (continuous lines) and Gank-S6-C complex (dashed line). Distributions are obtained via kernel density estimation analysis with Gaussian bandwidth of 1.6 ns.

The ankyrin repeats of Gank unfolded sequentially, one after the other, as judged by the RMSD from the native structure. Successive repeat unfolding events were evenly spaced in time for both uncomplexed and complexed Gank (Figure 7). The presence of S6-C bound to Gank introduced delays in unfolding, which were more pronounced for the later repeat unfolding events.

The precise sequence of unfolding of the repeats carries important information about the distribution of mechanical stability across the repeat array. For isolated Gank the most common unfolding sequence was from the C-terminus to the N-terminus although several other unfolding pathways are also observed (Table 1 top and Figure S2 top). Such behavior is expected for repeat proteins because the sequence similarity between the repeats means that they all have similar stabilities, which introduces degeneracy into the unfolding mechanism. The presence of bound S6-C had a dramatic impact on the sequence of unfolding (Figure S2 bottom); for example, repeat 1 (r1) is rarely the last repeat to unfold in complexed Gank. Overall, the key feature of the different behavior is the narrower spectrum of unfolding positions available to repeats r1, r2, r5 and r6 in complexed Gank. A quantitative comparison of the unfolding sequences in the complexed *versus* uncomplexed Gank can be performed using contingency tables built by counting the number of simulation runs where repeat r1 has a certain position in the unfolding sequence in complexed *versus* uncomplexed Gank (Table 1). An exact Fisher's test on this contingency table shows that complexed Gank has a significantly different unfolding sequence compared with uncomplexed Gank (confidence level 0.006%). Similarly significant differences (maximum confidence level 2.3%) are obtained using repeats r2, r5 or r6. We built these contingency tables by pooling all the simulations at both pulling speeds as the same kind of test revealed that the unfolding sequences were not significantly dependent on the pulling speed. The change in the sequence of repeat unfolding events and the time delays in the later unfolding events results in an increase in the average unfolding time of repeats r1–r4 in the complex compared with isolated Gank (Tables S3 and S4). In summary, the data show that S6-C binding significantly modulates the forced unfolding of Gank by specifically stabilizing the ligand-contacting repeats, which results in prolonged lifetimes of these repeats and less diversity of unfolding pathways accessible to the protein.

**Table 1:** Order of repeat unfolding.

Uncomplexed Gank							
	1st	2nd	3rd	4th	5th	6th	7th
r1	0	6	7	3	0	4	8
r2	0	0	3	3	5	11	6
r3	0	0	0	2	13	5	8
r4	0	0	1	11	5	6	5
r5	0	1	14	7	4	2	0
r6	0	21	3	2	1	0	1
r7	28	0	0	0	0	0	0

Gank-S6-C complex							
	1st	2nd	3rd	4th	5th	6th	7th
r1	0	0	3	16	4	10	3
r2	0	0	0	1	9	8	18
r3	0	0	0	0	14	15	7
r4	0	0	0	17	9	2	8
r5	0	0	33	2	0	1	0
r6	0	36	0	0	0	0	0
r7	36	0	0	0	0	0	0

Entries are the number of SMD runs in which the repeat indicated unfolds first, second, third etc. The data from simulations at the two pulling speeds were pooled.

**Figure 8:**
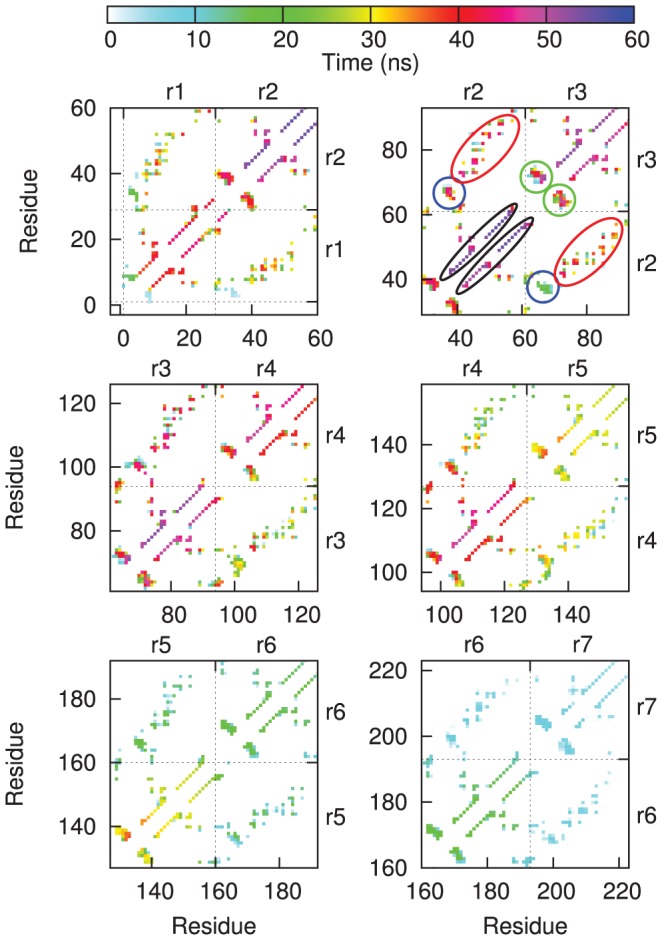
Contact map of Gank showing the lifetime of native C_α_-C_α_ contacts between residues observed in the isolated Gank (lower diagonal) and Gank-S6-C complex (upper diagonal) simulations at 0.01 Å/ps pulling speed. The contact map is subdivided in six panels showing data for the pairs of adjacent repeats (repeat numbers are reported on the left and top side of the panels). The color shade of each contact indicates the time the contact was last consistently observed along the simulations, averaged over the runs (see Materials and Methods). Colored ellipses in the top right panel indicate relevant groups of contacts, which can then be located correspondingly in the other panels: intra-repeat helix contacts (black ellipses), inter-repeat helix contacts (red ellipses), intra-repeat β-turn contacts (green ellipses) and inter-repeat β-turn contacts (blue ellipses).

A more detailed analysis of the force-induced unfolding process is obtained by monitoring the lifetime of native contacts in Gank (Figure 8). The lifetime of intra-repeat contacts (Figure 8, the α-helical contacts and the β-turn contacts within the black and the green ellipses, respectively) confirms the directional character of the unfolding process, which proceeds roughly from the C- to the N-terminus, and the larger resistance of the complexed Gank compared with the isolated Gank. A prominent difference between the two sets of simulations is the early loss of inter-repeat contacts between β-turn regions (within the blue ellipses in Figure 8) observed for repeat pairs r2–r3, r3–r4 and r4–r5 in isolated Gank and not in the Gank-S6-C complex. In contrast, inter-repeat helix-helix contacts (red ellipses) tend to be lost just before one of the two repeats of the pair unfolds.

The non-native contacts observed in the simulations are mostly local in nature, i.e., occurring between residues close in sequence, and they are slightly more frequent in uncomplexed Gank than in the complex (Figure S3). A closer inspection of the data reveals that they involve the force-induced deformations of the repeat array, whereby one repeat rotates with respect to the adjacent repeat around the long axis of the protein.

### Long-lasting interactions under mechanical stress map out the binding hot spots

**Figure 9:**
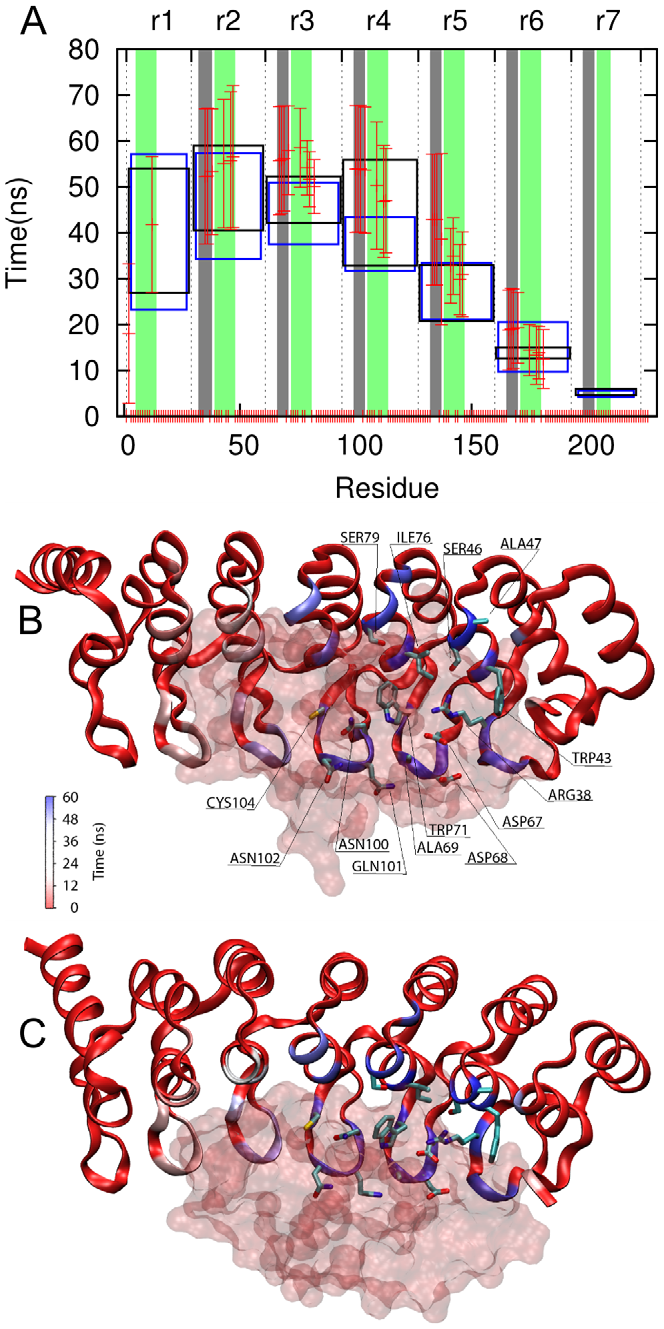
Time dependence of the binding contacts between Gank and S6-C at 0.01 Å/ps pulling speed. A. Lifetime of binding contacts with S6-C for the residues of Gank (red error bars represent 1 standard error around average). Black and blue boxes represent 1 standard error around the average unfolding time of the corresponding repeats in Gank-S6-C complex and isolated Gank simulations, respectively, as measured using RMSD. Grey and green shadowed regions indicate the beta-turn and N-terminal helix regions, respectively. (B)–(C). Front and upper views of Gank (cartoon representation), colored according to the average time each residue took to lose contact with S6-C (glass surface representation, displayed to mark the position of the binding site in the native conformation). The residues that form long-lasting contacts with S6-C are shown in licorice. The figure was prepared using VMD [38].

The simulations of the Gank-S6-C complex show how specific features of the interaction between the two proteins are affected by the mechanical stress exerted on Gank. The binding surface in the unperturbed complex comprises residues from repeats r1–r6 of Gank. Most of the contacting residues are in the β-turn and the N-terminal α-helix of each repeat. The loss of contacts between the two proteins during the simulations occurs gradually and follows the sequence of unfolding of the repeats themselves (Figure 9A). The contacts that fail last are those involving residues in repeats r2, r3 and r4, more specifically the β-turn of repeat 4, the β-turn and N-terminal α-helix of repeat 3 and the N-terminal α-helix of repeat r2 (Figure 9B and C and Table 2). The large variability in the lifetime of binding contacts in repeats r2 and r4 is related to the variability in the observed unfolding sequence in the different simulation runs (Figure S2), whereby one of the two repeats is most often the last to unfold. Within repeats 4–6, the lifetimes of the binding contacts involving residues in the β-turn are slightly larger than those in the N-terminal α-helix (Figure 9A). Binding contacts involving residues in repeat 3 tend to survive the unfolding of the repeat, specifically residues Ile76 and Trp71 in the N-terminal α-helix of repeat 3. These interactions appear to have a non-specific hydrophobic character that does not require a perfectly structured repeat to be maintained.

**Table 2:** Gank residues involved in long-lived contacts with ligand S6-C.

Repeat	Residues
r2	Arg38, Trp43, Ser46, Ala47
r3	Asp67, Asp68, Ala69, Trp71, Ile76, Ser79
r4	Asn100, Gln101, Asn102, Cys104

## Discussion

### Effects of ligand binding on the mechanical stability of repeat proteins

Ligand binding typically increases the thermodynamic stability of proteins, whereas mechanical stabilization upon ligand binding has been observed for some proteins [11], [13], [14], [26]–[28] but not for others [12], [29], [30]. This distinction arises because mechanical unfolding as measured by force microscopy using a constant pulling speed is not an equilibrium process and therefore mechanical stability reflects the height of the energy barrier, i.e. the difference in stability between the native state and the mechanical transition state. Moreover, force-induced unfolding may often be very different from chemical-induced unfolding because of the fundamental difference between the two reaction coordinates and the different nature of the unfolded states in the two reactions. A ligand will enhance the mechanical stability of a protein only if it preferentially stabilizes the native state over the mechanical unfolding transition state, an approach that has been used to rationally tune mechanical stability [14]. The situation is more complicated for repeat proteins, however, because mechanical unfolding proceeds in a stepwise manner and so there is a series of transition states rather than a single one. We show here that ligand binding to a repeat protein has a discrete effect on its mechanical stability that is localized to specific repeats and consequently it dramatically alters the protein's mechanical behavior. The bound ligand increases the resistance of Gank to mechanical stress by delaying its complete unfolding and by requiring larger forces and consequently a larger energy to unfold it. We speculate that the ligand offers a supplemental support frame (or “sink”) to the protein that helps it to withstand the external force by distributing the stress over a larger molecular volume.

The high pulling speeds used in simulations have been shown in some cases to mask the differences between two systems under comparison, due to the high noise level [31]. However, the differences we observed between complexed and uncomplexd Gank are insensitive to changes in the pulling speed and they are significant even at the high pulling speeds of the simulations; we predict therefore that significant differences should be observed experimentally in force-extension profiles measured by AFM. Indeed, our simulations predict up to two-fold difference in the forces observed for complexed *versus* uncomplexed Gank independent of pulling speed. And since the unfolding force peaks of isolated ankyrins are experimentally detectable [3], [5], [6], so should be the detection of higher force peaks for upon complexation.

### Fewer unfolding pathways are accessible to repeat proteins upon ligand binding

We found previously, and confirmed in the present study, that when subjected to an external pulling force the predominant unfolding pathway of Gank is from the C-terminus to the N-terminus [6], although a variety of other unfolding pathways are also accessible. This behavior is profoundly altered in the presence of the ligand. One consequence of ligand binding is that the N-terminal repeat (r1) rarely unfolds last, which also explains the large change in the r1 component of transferred work: indeed, unfolding of a repeat with a folded neighbor (r2 in this case) requires extra energy due to the inter-repeat interactions. More generally, we observe that ligand binding greatly reduces the number of unfolding routes accessible to Gank, and this can be rationalized as follows: The sequence similarity between the repeats means that they all have similar stabilities and therefore unfolding can proceed through many different pathways. Indeed multiple pathways have also been observed in chemical-induced unfolding of repeat proteins (e.g. [32]); Ligand binding stabilizes repeats r2–r5 (the mechanical hot spots), making the stability distribution across the repeat array more uneven and resulting in energetic bias and less diversity of unfolding pathways.

### Cooperativity in repeat-protein mechanical unfolding changes upon ligand binding

The bound ligand induced a more regular and cooperative mechanical unfolding behavior at the repeat level, in the sense that isolated Gank showed a wide variety of stretching patterns involving both partially unfolded repeats, whereby only one of the two helices is unfolded, and the unfolding of more than one repeat at a time (Figure 5, top panel); in contrast, the S6-C-bound Gank tended to unfold more “sharply” repeat by repeat (Figure 5, lower panel), similar to the consensus ankyrin repeat protein NI_3_C [6], [23]. S6-C binding has the effect of reducing the importance of the cross talk between repeats by compensating for the loss of inter-repeat stability upon unfolding with the stabilization of each individual repeat involved in binding (particularly the β-turns regions as shown in Figure 8).

### Mapping binding hot spots in tandem repeat proteins

The simulations helped to identify those residues of Gank whose contacts with S6-C are the most resilient to pulling (Figure 9B, C and Table 2). These residues are Arg38, Trp43, Ser46, Ala47, Asp67, Asp68, Ala69, Trp71, Ile76, Ser79, Asn100, Gln101, Asn102, Cys104 (according to our numbering scheme). We can compare these mechanical hot spots with the thermodynamic hot spots investigated by pull-down experiments [9]. Pull-downs were used to test mutations at positions Arg38, Lys113, Asp36, Asp68 and Glu159 (according to our numbering scheme), but only mutation of Arg38 was found to disrupt S6-C binding sufficiently to prevent pull down. The other residues did not appear to make as big a contribution to the interaction, which agrees with our finding that they do not have a large contact lifetime with S6C in our simulations. Contrary to the pull-down results, we found that Asp68 had a high contact lifetime. This may be a consequence of the fact that both neighboring residues Ala69 and Asp67 have large contact lifetimes, rather than its role in binding.

### Implications of ligand binding for mechanical signal transduction in repeat proteins

The mechanical unfolding of repeat proteins is characterized by relatively small force peaks; therefore, the force at rupture is determined with a much larger relative error than in less compliant systems, making it more difficult to detect a trend in the height of the force peak as a function of the unfolding event as predicted by [33]. Nevertheless, the simulations do clearly show two distinct force regimes of complexed Gank: up to 300 Å total extension, corresponding most frequently to the unfolding of repeats 7 and 6, the forces were similar to those observed in uncomplexed Gank. Above 300 Å total extension, corresponding most frequently to the unfolding of repeats 1–5, the unfolding forces were much higher than those at lower extensions. This property of repeat proteins might have an important functional role in mechanotransduction, by preventing the complete dissociation of a protein complex below a certain force threshold. A mechano-switch can be thought of as a switch between two shapes of the free energy that rules thermodynamics, kinetics and the modulation of the latter with a force. Repeat proteins composed of units with unfolded contour lengths ranging between ∼100–150 Å, such as ankyrin repeat proteins and HEAT repeat proteins, could function as mechano-switches because their repeats are progressively unfolded only with increasing forces [33], thereby allowing mechanical stress to modulate the protein structure and its binding properties. Moreover, the elastic behavior of the individual repeats [6] could contribute to the stability of repeat protein complexes by allowing refolding of individual repeats before complete dissociation of the protein from its ligand. The different force regimes under which Gank unfolds when bound to S6-C might have relevance in the mechanical control of protein-protein interactions in the cell and this behavior could be exploited in future to design new protein mechano-switches.

The characteristic stepwise mechanical unfolding of repeat proteins is at the very heart of their compliance and an essential component of their elastic behavior [1], [2]. These properties implicate repeat proteins in mechanical signal transduction. For example: repeat-protein stretching and contraction motions to regulate the activity of a bound enzyme [34]; ankyrin nanosprings to operate the gating of ion channels in hair cells [35]; HEAT-repeat carrier proteins that wrap around their cargoes to transport them between the cytoplasm and the nucleus [36]. The sensitivity of repeat-protein mechanics to ligand binding, as revealed in the present work, suggests the possibility of a further regulatory mechanism whereby elasticity is dictated by ligand binding.

## Supporting Information

Figure S1
**Work transferred to the system by the pulling force.** Representative examples of the time dependence of the repeat components (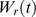, see Materials and Methods) of the work transferred by the pulling force from simulations of isolated Gank (two top panels) and Gank-S6-C complex (two lower panels) at 0.01 Å/ps pulling speed.(EPS)

Figure S2 **Unfolding time of each ankyrin repeat of Gank in the various simulation runs at 0.01 Å/ps pulling speed.** Data for isolated Gank and the Gank-S6-C complex are shown in the upper and lower boxes, respectively. Data from the different repeats are shown in different colors. The runs are ordered on the x-axis so that similar repeat unfolding sequences are adjacent. The lines connecting the points are drawn only as a guide for the eye. Isolated Gank unfolds most frequently in a sequential manner from the C- to the N-terminal repeat (right part of top box). For Gank in the complex, in contrast, the last repeats to unfold are frequently repeats 2, 3 and 4.
(EPS)

Figure S3 **Contact map showing all of the observed recurrent contacts between C_α_ atoms (see** **Materials and Methods** **).** Data for isolated Gank and the Gank-S6-C complex at 0.01 Å/ps pulling speed are reported in the lower and upper diagonals, respectively. The color of each contact reports the fraction of the simulation runs where it was observed. Thus, native contacts (the most frequent) appear usually in dark blue. A larger fraction of non-native contacts are observed in isolated Gank between R1 and R2 and between R2 and R3, than those observed in the complex.
(TIF)

Table S1 **Comparison of average repeat components of transferred work (in K_b_T) at 0.01 Å/ps pulling speed for uncomplexed and complexed Gank.**
(DOC)

Table S2 **Comparison of average repeat components of transferred work (in K_b_T) at 0.05 Å/ps pulling speed for uncomplexed and complexed Gank.**
(DOC)

Table S3 **Comparison of average repeat unfolding times (in ns) at 0.01 Å/ps pulling speed for uncomplexed and complexed Gank.**
(DOC)

Table S4 **Comparison of average repeat unfolding times (in ns) at 0.05 Å/ps pulling speed for uncomplexed and complexed Gank.**
(DOC)

Text S1 **Definition of recurrent contacts.**
(DOC)
