# Supplementary figures and images for: Effects of Ligand Binding on the Mechanical Properties of Ankyrin Repeat Protein Gankyrin
Source: PLoS Comput Biol. 2013 Jan 17;9(1):e1002864. doi: 10.1371/journal.pcbi.1002864 (PMC3547791; doi:10.1371/journal.pcbi.1002864)

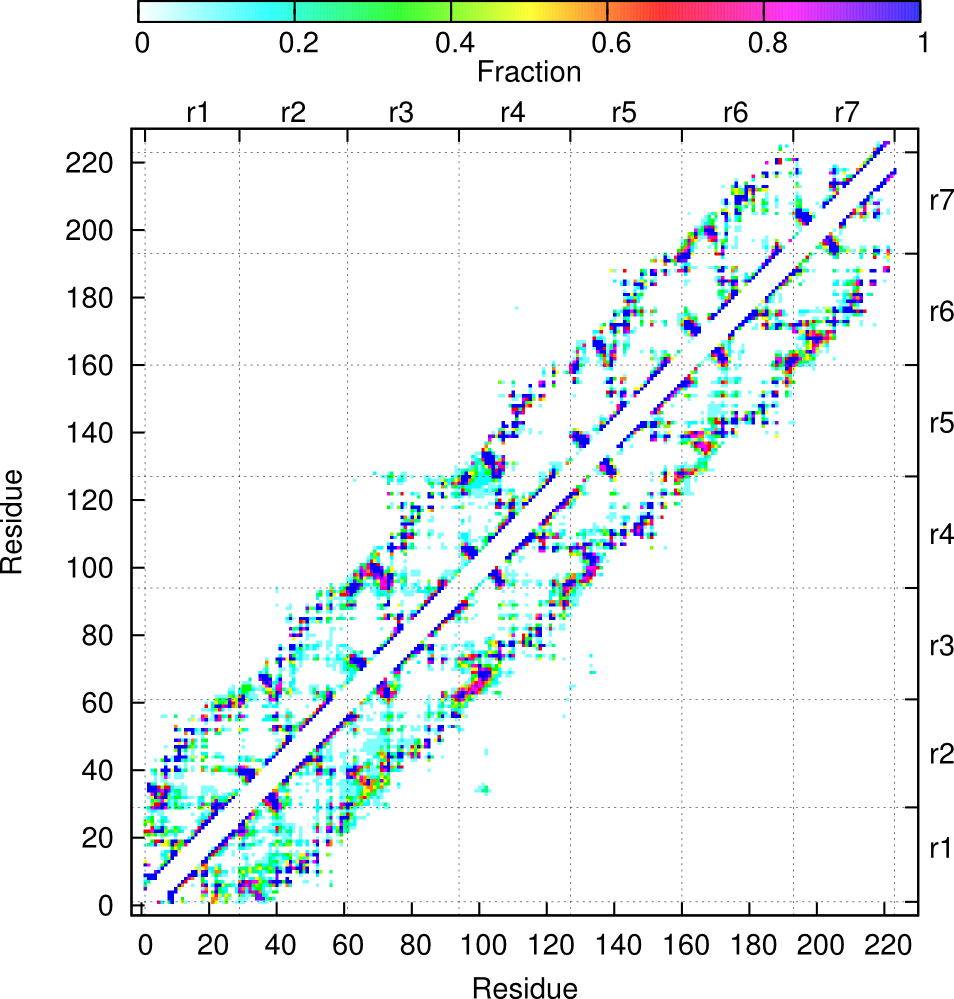

Supplement: Figure S3 — Contact map showing all of the observed recurrent contacts between Cα atoms (see Materials and Methods ). Data for isolated Gank and the Gank-S6-C complex at 0.01 Å/ps pulling speed are reported in the lower and upper diagonals, respectively. The color of each contact reports the fraction of the simulation runs where it was observed. Thus, native contacts (the most frequent) appear usually in dark blue. A larger fraction of non-native contacts are observed in isolated Gank between R1 and R2 and between R2 and R3, than those observed in the complex. (TIF) [file pcbi.1002864.s003.tif]
