# Supplementary material for: Effects of Ligand Binding on the Mechanical Properties of Ankyrin Repeat Protein Gankyrin
Source: PLoS Comput Biol. 2013 Jan 17;9(1):e1002864. doi: 10.1371/journal.pcbi.1002864 (PMC3547791; doi:10.1371/journal.pcbi.1002864)
Supplement: Table S1 — Comparison of average repeat components of transferred work (in KbT) at 0.01 Å/ps pulling speed for uncomplexed and complexed Gank. (DOC) [file pcbi.1002864.s004.doc]

**Table S1. Comparison of average repeat components of transferred work (in K_b_T) at 0.01 Å/ps pulling speed for uncomplexed and complexed Gank.**

| **Repeat** | **Gank-S6C** | **Uncomplexed Gank** | **Ratio** |
| --- | --- | --- | --- |
| **r1** | 280 ± 20 | 130 ± 10 | 2.15 |
| **r2** | 340 ± 30 | 170 ± 10 | 1.93 |
| **r3** | 330 ± 20 | 210 ± 20 | 1.59 |
| **r4** | 310 ± 20 | 230 ± 10 | 1.34 |
| **r5** | 420 ± 20 | 240 ± 10 | 1.76 |
| **r6** | 210 ± 20 | 240 ± 10 | 0.86 |
| **r7** | 240 ± 10 | 160 ± 20 | 1.54 |
