# Supplementary material for: Effects of Ligand Binding on the Mechanical Properties of Ankyrin Repeat Protein Gankyrin
Source: PLoS Comput Biol. 2013 Jan 17;9(1):e1002864. doi: 10.1371/journal.pcbi.1002864 (PMC3547791; doi:10.1371/journal.pcbi.1002864)
Supplement: Table S2 — Comparison of average repeat components of transferred work (in KbT) at 0.05 Å/ps pulling speed for uncomplexed and complexed Gank. (DOC) [file pcbi.1002864.s005.doc]

**Table S2. Comparison of average repeat components of transferred work (in K_b_T) at 0.05 Å/ps pulling speed for uncomplexed and complexed Gank.**

| **Repeat** | **Gank-S6C** | **Uncomplexed Gank** | **Ratio** |
| --- | --- | --- | --- |
| **r1** | 410 ± 10 | 230 ± 10 | 1.76 |
| **r2** | 460 ± 10 | 370 ± 10 | 1.23 |
| **r3** | 570 ± 20 | 340 ± 10 | 1.67 |
| **r4** | 580 ± 20 | 380 ± 10 | 1.51 |
| **r5** | 510 ± 10 | 390 ± 10 | 1.30 |
| **r6** | 390 ± 10 | 350 ± 10 | 1.11 |
| **r7** | 310 ± 10 | 240 ± 10 | 1.25 |
