# Supplementary material for: Effects of Ligand Binding on the Mechanical Properties of Ankyrin Repeat Protein Gankyrin
Source: PLoS Comput Biol. 2013 Jan 17;9(1):e1002864. doi: 10.1371/journal.pcbi.1002864 (PMC3547791; doi:10.1371/journal.pcbi.1002864)
Supplement: Table S3 — Comparison of average repeat unfolding times (in ns) at 0.01 Å/ps pulling speed for uncomplexed and complexed Gank. (DOC) [file pcbi.1002864.s006.doc]

**Table S3. Comparison of average repeat unfolding times (in ns) at 0.01 Å/ps pulling speed for uncomplexed and complexed Gank.**

| **Repeat** | **Gank-S6C** | **Uncomplexed Gank** | **Difference** |
| --- | --- | --- | --- |
| **r1** | 40.5 ± 1.1 | 40.2 ± 1.4 | 0.28 |
| **r2** | 49.8 ± 0.8 | 45.8 ± 1.0 | 3.93 |
| **r3** | 47.2 ± 0.4 | 44.2 ± 0.6 | 3.00 |
| **r4** | 44.4 ± 1.0 | 37.5 ± 0.5 | 6.81 |
| **r5** | 26.8 ± 0.5 | 27.3 ± 0.5 | -0.40 |
| **r6** | 13.8 ± 0.1 | 15.1 ± 0.5 | -1.31 |
| **r7** | 5.3 ± 0.1 | 4.9 ± 0.1 | 0.39 |
