# Supplementary material for: Effects of Ligand Binding on the Mechanical Properties of Ankyrin Repeat Protein Gankyrin
Source: PLoS Comput Biol. 2013 Jan 17;9(1):e1002864. doi: 10.1371/journal.pcbi.1002864 (PMC3547791; doi:10.1371/journal.pcbi.1002864)
Supplement: Table S4 — Comparison of average repeat unfolding times (in ns) at 0.05 Å/ps pulling speed for uncomplexed and complexed Gank. (DOC) [file pcbi.1002864.s007.doc]

**Table S4. Comparison of average repeat unfolding times (in ns) at 0.05 Å/ps pulling speed for uncomplexed and complexed Gank.**

| **Repeat** | **Gank-S6C** | **Uncomplexed Gank** | **Difference** |
| --- | --- | --- | --- |
| **r1** | 8.26 ±  0.09 | 6.39 ±  0.20 | 1.87 |
| **r2** | 11.04 ±  0.06 | 8.68 ±  0.15 | 2.36 |
| **r3** | 10.29 ±  0.04 | 9.32 ±  0.10 | 0.97 |
| **r4** | 8.74 ±  0.08 | 8.46 ±  0.12 | 0.28 |
| **r5** | 5.25 ±  0.02 | 5.80 ±  0.09 | -0.54 |
| **r6** | 3.12 ±  0.01 | 3.78 ±  0.10 | -0.65 |
| **r7** | 1.27 ±  0.01 | 1.16 ±  0.01 | 0.11 |
