# Supplementary material for: Effects of Ligand Binding on the Mechanical Properties of Ankyrin Repeat Protein Gankyrin
Source: PLoS Comput Biol. 2013 Jan 17;9(1):e1002864. doi: 10.1371/journal.pcbi.1002864 (PMC3547791; doi:10.1371/journal.pcbi.1002864)
Supplement: Text S1 — Definition of recurrent contacts. (DOC) [file pcbi.1002864.s008.doc]

**Supporting Information**

**Definition of recurrent contacts**

For each contact *i* we have measured the following quantity along the trajectory:

| $C_{i}(t_{N})=\sum_{t=0}^{t_{N}} e^{\frac{1}{\tau}(t_{N}-t)}c_{i}(t)$ | **(SI 1),** |
| --- | --- |

where *c_i_*(*t*) is 1 or 0 if the contact is present or not at time *t*, respectively, and τ is a decay time, which we set to 0.2 ns as a reasonable time window for expecting a stable atomic contact to remain formed. Eq. SI 1 can be viewed as a running average where past time snapshots have a weight that decays exponentially with time distance. If a contact *i* is always present, then $C_{i}(t_{N})\approx\frac{1}{1-e^{-\frac{1}{\tau}}}=F$is independent from the time *t_N_*. Recurrent contacts are those where $C_{i}(t_{N})\geq F/2$ at least once along the trajectory. This is broadly equivalent to observing that the contact is present in half of the snapshots of a 0.4 ns time window (approximately the time window before *t_N_* where the weight in Eq. SI 1 is larger than 0).
